# A comparative study of interhemispheric functional connectivity in patients with basal ganglia ischemic stroke

**DOI:** 10.3389/fnagi.2024.1408685

**Published:** 2024-09-25

**Authors:** Jian Zhang, Shijian Chen, Chengmin Yang, Huo Liang, Xuemei Quan, Yayuan Liu, Zhijian Liang

**Affiliations:** ^1^Department of Neurology, The Second Affiliated Hospital of Guangxi Medical University, Nanning, Guangxi, China; ^2^Department of Neurology, The Fourth Affiliated Hospital of Guangxi Medical University, Liuzhou, Guangxi, China; ^3^Department of Neurology, Affiliated Hospital of Youjiang Medical University for Nationalities, Baise, Guangxi, China; ^4^Department of Neurology, The First Affiliated Hospital of Guangxi Medical University, Nanning, Guangxi, China; ^5^Department of Neurology, The People’s Hospital of Guangxi Zhuang Autonomous Region, Nanning, Guangxi, China; ^6^Department of Neurology, The Affiliated Minzu Hospital of Guangxi Medical University, Nanning, Guangxi, China

**Keywords:** voxel-mirrored homotopic connectivity, functional connectivity, basal ganglia ischemic stroke, resting-state functional magnetic resonance imaging, stroke

## Abstract

**Background:**

Voxel-mirrored homotopic connectivity (VMHC) is utilized to assess the functional connectivity of neural networks by quantifying the similarity between corresponding regions in the bilateral hemispheres of the brain. The exploration of VMHC abnormalities in basal ganglia ischemic stroke (BGIS) patients across different cerebral hemispheres has been limited. This study seeks to establish a foundation for understanding the functional connectivity status of both brain hemispheres in BGIS patients through the utilization of VMHC analysis utilizing resting-state functional magnetic resonance imaging (rs-fMRI).

**Methods:**

This study examined a total of 38 patients with left basal ganglia ischemic stroke (LBGIS), 44 patients with right basal ganglia ischemic stroke (RBGIS), and 41 individuals in a healthy control (HC) group. Rs-fMRI studies were performed on these patients, and the pre-processed rs-fMRI data were analyzed using VMHC method. Subsequently, the VMHC values were compared between three groups using a one-way ANOVA and *post hoc* analysis. Correlation analysis with clinical scales was also conducted.

**Results:**

The results indicated that compared to the HC group, significant differences were detected in postcentral gyrus, extending to precentral gyrus in both BGIS groups. *Post hoc* analysis showed that in the pairwise ROI-based comparison, individuals with LBGIS and RBGIS exhibited reduced VMHC values compared to HC groups. There was no significant difference between the LBGIS and RBGIS groups. In the LBGIS group, the VMHC value showed a negative correlation with NIHSS and a positive correlation with BI.

**Conclusion:**

The analysis of VMHC in rs-fMRI revealed a pattern of brain functional remodeling in patients with unilateral BGIS, marked by reduced synchronization and coordination between hemispheres. This may contribute to the understanding of the neurological mechanisms underlying motor dysfunction in these patients.

## Introduction

Stroke, now the second leading cause of mortality globally, presents significant detrimental consequences that warrant attention. It is typically categorized into ischemic and hemorrhagic stroke, with ischemic stroke prevailing at a rate of 69.6–70.8% of all cases (Wang D. et al., 2017; Wang W. et al., 2017). The basal ganglia, situated in the deep regions of the brain, serves as a critical site commonly affected by ischemic stroke (Araki et al., 1983). Given its significance as a pivotal hub for pyramidal tract conduction and its role in motor execution and control, preserving the functional and structural integrity of this area is essential for sustaining typical motor function (Gómez-Ocádiz and Silberberg, 2023). As a result, ischemic stroke affecting the basal ganglia commonly presents with symptoms such as severe contralateral limb movement impairments, significantly impacting their overall quality of life (Kaas and Stepniewska, 2023; Florio et al., 2018). Furthermore, research has identified potential correlations between the basal ganglia and language function (Radanovic and Mansur, 2017), cognitive function (Zhao et al., 2018), depression (Wu et al., 2014) and post-stroke fatigue (Tang et al., 2010). Consequently, investigating the stroke severity, motor function, ability of daily living, cognitive function, and depressive status of basal ganglia ischemic stroke (BGIS) holds significant importance and may offer novel perspectives for future studies on the functional rehabilitation of individuals affected by BGIS.

Within the intricate context described, resting-state functional magnetic resonance imaging (rs-fMRI) has garnered significant interest in the scientific community as an emerging method (Wei et al., 2024). Voxel-mirrored homotopic connectivity (VMHC), a special rs-fMRI analysis technique that leverages voxel mirror symmetry, has also garnered considerable attention from researchers (Zuo et al., 2010). The method of VMHC is utilized to assess the functional connectivity of neural networks by quantifying the similarity between corresponding regions in the bilateral hemispheres of the brain (Zuo et al., 2013). This novel approach offers a fresh insight into comprehending the etiology of stroke. Specifically, VMHC has the capability to depict the functional connection status among diverse brain regions, with alterations in this connectivity potentially playing a significant role in the development of ischemic stroke (Tang et al., 2016; Chen et al., 2021). Secondly, previous study has identified significant deviations in the parameters of VMHC in stroke patients, including diminished functional connection strength and alterations in network topology structure, potentially linked to the pathogenesis of ischemic stroke (Li et al., 2022). Furthermore, research indicates that modifications in VMHC parameters have the potential to forecast the prognosis of stroke and offer valuable guidance for treatment strategies (Shan et al., 2018).

Given the limited exploration of functional connectivity abnormalities between BGIS patients of varying hemispheres in prior research (Yao et al., 2020), the utilization of VMHC as a potential innovative tool for diagnosis and evaluation of therapeutic effects is anticipated. Our hypothesis posits that following BGIS, there will be alterations in the functional connectivity pattern of homologous brain regions across the two cerebral hemispheres, potentially influencing clinical outcomes. The objective of this study was to enhance comprehension of alterations in interhemispheric functional coordination in unilateral BGIS patients through an analysis of mirror homotopy connection patterns in patients with ischemic stroke affecting distinct hemispheres, and to explore its association with clinical symptoms. BGIS patients were stratified into two cohorts according to the hemisphere of stroke, and a control group of healthy individuals was recruited for comparative analysis with the BGIS patients.

## Materials and methods

### Participants

The study enrolled a cohort of first-time acute BGIS patients and healthy volunteers from May 2019 to October 2022, with all BGIS patients being hospitalized in the Neurology Department of the First Affiliated Hospital of Guangxi Medical University, while healthy volunteers were recruited from neighboring communities, hospital health workers, and family members of patients through advertisements. This study received approval from the Ethics Committee of the First Affiliated Hospital of Guangxi Medical University [ethics approval number 2021 (KY-E-184)], and all participants provided written informed consent prior to the commencement of the study. The objectives and importance of the study were communicated to all participants, and all procedures were conducted in strict adherence to the clinical trial protocol.

#### Inclusion criteria for the acute BGIS group

(1) Age: between 18 and 80 years old; (2) Time from onset to MRI examination within 1 week; (3) Diagnosis of acute ischemic stroke conforms to “Guidelines for the Diagnosis and Treatment of Acute Ischemic Stroke in China (2018),” diagnosed by a neurology resident physician and an attending or higher-level physician through medical history collection, physical examination, and imaging examination; (4) Ischemic stroke confirmed by MRI examination as first-time ischemic stroke, located in the basal ganglia region, no other intracranial lesions except for the stroke lesion; (5) The patient is conscious and voluntarily agrees to cooperate with MRI examination and scale assessment; (6) No contraindications for MRI examination; (7) No history of depression or other mental disorders before onset; (8) No history of alcohol or drug dependence; (9) Right-handedness confirmed by the Edinburgh Handedness Questionnaire.

#### Exclusion criteria for the acute BGIS group

(1) Any contraindications for MRI examination such as cardiac pacemaker implantation, internal metal implantation, claustrophobia; (2) Pregnant or lactating women; (3) Other old ischemic stroke or tumors, brain injuries, demyelinating diseases found by MRI examination after onset; (4) Severe physical diseases such as heart, liver, kidney, head injury history, epilepsy history, and mental disorders; (5) During MRI examination, excessive head movement range (subjects who move in any direction of x, y, z more than 3.0 mm or head rotation angle > 3° during scanning) causes poor image data or incomplete data that affects subsequent analysis; (6) White matter high signal intensity > 1 point determined by professional imaging physicians according to the modified Fazekas score; (7) Nii image with bad normalization after data preprocessing.

#### Inclusion criteria for the healthy control (HC) group

(1) Age: between 18 and 80 years old; (2) No contraindications for MRI examination; (3) Age and education level match those of the patient group; (4) No history of myocardial infarction, stroke, or other serious systemic diseases.

#### Exclusion criteria for the HC group

Same as acute BGIS group exclusion criteria.

### Neurological scale scores

The National Institute of Health Stroke Score (NIHSS) was utilized to evaluate the severity of stroke in patients with acute BGIS during the hospitalization. The Barthel Index (BI) was employed to assess the daily living capabilities of these patients, while the Fugl-Meyer assessment (FMA) was used to assess their motor function. The Montreal Cognitive Assessment (MoCA) was implemented to evaluate the cognitive function status, and the self-rating depression scale (SDS) was used to determine the degree of post-stroke depression experienced by these patients.

### MRI data collection

MRI data collection was performed using a Siemens Prisma 3.0T MRI scanner at the Department of Radiology, the First Affiliated Hospital of Guangxi Medical University. All participants were instructed to lie still in a supine position during the scanning process with their eyes open and without moving their body. T1-weighted imaging (T1WI) sequence was obtained as conventional MRI sequence.

High-resolution three-dimensional sagittal T1-weighted images of the whole brain were acquired using the 3D BRAVO sequence. The scanning parameters were as follows: repetition time (TR) = 2,300 ms, echo time (TE) = 2.98 ms, inversion time = 900 ms, slice thickness = 1 mm, voxel size = 1 mm × 1 mm × 1 mm, interslice gap = 0 mm, field of view (FOV) = 256 mm × 256 mm, and 176 sagittal images were obtained. The scanning time was 5 min and 21 s.

The rs-fMRI was performed using echo-planar imaging (EPI) sequence axial scanning. The scanning parameters were as follows: TR/TE = 2,000/35 ms, slice thickness = 3 mm, voxel size = 2.6 mm × 2.6 mm × 3 mm, number of slices = 40, matrix = 64 × 64, FOV = 240 mm × 240 mm, flip angle (FA) = 90°, and the scanning time was 6 min and 20 s.

All participants underwent the same MRI scanning sequences and parameters.

### Lesion map

**FIGURE 1:**
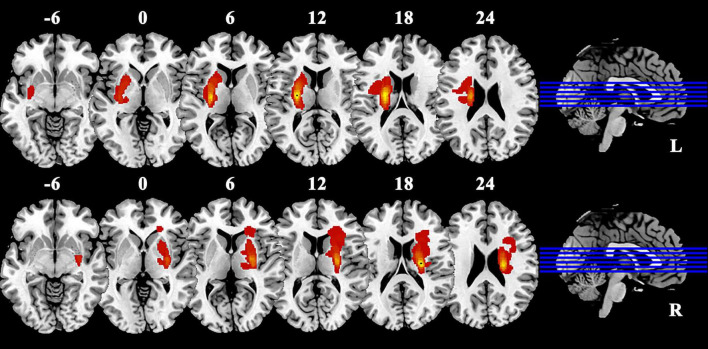
Lesion overlap in patients with BGIS. Warm colors represent the spatial co-localization of pathological regions within individual BGIS patients. BGIS, basal ganglia ischemic stroke; L, left; R, right.

We utilized T1 images for conducting lesion imaging of patients with BGIS (Figure 1). The lesion site of each subject was meticulously delineated using MRIcron software (Rorden et al., 2007). The anatomical structure and contours of the lesion can be distinctly identified in the T1 images. Two neurologists independently marked the stroke location on the T1 images. By aligning and merging the individual focal masks, we obtained spatially normalized focal masks specifically for BGIS patients.

### fMRI data preprocessing

The rs-fMRI image data preprocessing in this study was performed using the Matlab (MathWorks, R2022a) platform with DPABI (Data Processing & Analysis for Brain Imaging, version 7.0) (Yan et al., 2016) tool based on SPM12 (Statistical Parametric Mapping, version 12) as well as xjview96 and BrainNet Viewer (Xia et al., 2013).

The rs-fMRI data preprocessing process includes: (1) Removing time points: To correct the image level time due to the need of subjects to adapt to the examination environment and eliminate the influence of the uneven magnetic field operation of the MRI machine at the beginning, remove the first 10 time point MRI image data; (2) Time layer correction: The scanning layer number is a total of 40 layers, with interval layer scanning, starting from the even layer, then scanning the odd layer; (3) Head movement correction: To reduce the noise caused by head movement during MRI scanning on signal, exclude subjects who move in any direction of x, y, z more than 3.0 mm or head rotation angle > 3° during scanning, according to this standard, 21 cases were excluded from the basal ganglia infarction group and 2 cases were excluded from the healthy control group; (4) Spatial normalization: Coregister structural image to the mean functional image after motion correction. Segment the transformed structural image into gray matter, white matter, cerebrospinal fluid by using a unified segmentation algorithm (New Segment). Spatially normalize the motion corrected functional volumes to the Montreal Neurological Institute (MNI) space (voxel size of 3 mm × 3 mm × 3 mm) using the normalization parameters estimated in DARTEL, so as to reduce the impact caused by differences in subject brain structure; (5) Interference covariate regression: To reduce the impact of white matter, cerebrospinal fluid signals, head movement (Friston-24 model) (Yan et al., 2013) on the results; (6) Remove linear drift: Remove the linear trend deviation caused by thermal noise of the preamplifier in front of the MRI machine coil; (7) Filtering: Apply a filter of 0.01–0.10 Hz to the signal.

### VMHC analysis

The VMHC was computed using the DPABI software package, which involved extracting the time series data from all voxels in one hemisphere of the subject’s brain, calculating the Pearson correlation coefficient between these time series and the mirrored voxel time series in the contralateral hemisphere to determine the VMHC value. Subsequently, a Fisher *z* transformation was applied to convert the Pearson correlation coefficient into a *z*-value to enhance normality, resulting in the generation of *z*-value maps of VMHC for each subject.

### Statistical analysis

SPSS 26.0 (Statistical Package for the Social Sciences, version 26.0) statistical software was used for demographic and clinical characteristics comparison. Normality tests were initially performed on numerical variables, with subsequent use of mean ± standard deviation and *t*-tests for inter-group comparisons if normality assumptions were met. In cases where normality assumptions were not met, median (quartile spacing) and nonparametric tests were utilized for statistical description and inter-group comparisons, respectively. Demographic data comparisons between groups were conducted using one-way ANOVA for age, education years, and other quantitative data, while chi-square tests were employed for gender and other categorical data comparisons.

The distribution of MoCA scores in the left/right BGIS groups adhered to a normal distribution, necessitating the use of an independent sample *t*-test for group comparison. Conversely, the NIHSS, FMA, BI, and SDS scores did not exhibit a normal distribution, thus requiring the utilization of the Mann–Whitney U test for comparison.

In the analysis of fMRI data, the zVMHC values of three subject groups were compared using a one-way ANOVA within the statistical module of the DPABI software package, with age, gender, and education level serving as covariates. Gaussian random field theory (GRF) was employed for multiple comparison correction. Significant differences were observed at the voxel *P* < 0.001 and cluster *P* < 0.05 (bilateral test), leading to the extraction of distinct brain regions. Brain regions with significant differences in VMHC values were defined as regions of interest (ROIs), and the mean VMHC values of these ROIs were extracted for *post hoc* analysis. We used ANOVA to investigate whether there were differences in the mean VMHC values among the three groups. Subsequently, the mean VMHC values were correlated with clinical scales using Pearson or Spearman correlation analysis in both BGIS groups. The statistical software R version 3.5.3 was used to perform *post hoc* analysis, correlation analysis and create visualizations. All tests were conducted as bilateral tests, with statistical significance defined as *P* < 0.05.

## Results

### Participants’ characteristics

A total of 192 subjects meeting the inclusion criteria were included in the study, with 107 in the BGIS group and 85 in the HC group. Four subjects from the BGIS group were excluded due to incomplete image data, while 23 subjects (21 from the BGIS group and 2 from the HC group) were excluded due to excessive head movement. Ultimately, 82 BGIS patients were included, with 38 having left BGIS (LBGIS) and 44 having right BGIS (RBGIS). Additionally, 41 subjects from the HC group were selected to match for age, gender, and education level, resulting in a total of 123 subjects included in the study. There were no statistically significant differences in gender, age, or years of education among the LBGIS group, the RBGIS group, and the HC group (*P* > 0.05). Additionally, there were no statistically significant differences in NIHSS, MoCA, BI, SDS, and FMA scores between the LBGIS group and the RBGIS group (*P* > 0.05) as shown in Table 1. The normality test revealed that NIHSS, BI, FMA and SDS scores did not adhere to a normal distribution, while age and MoCA scores did conform to a normal distribution.

**TABLE 1:** Demographic and clinical characteristics of the participants.

	LBGIS (*n* = 38)	RBGIS (*n* = 44)	HC (*n* = 41)	Test value	*P*-value
Age (years)^a^	58.08 ± 9.454	54.36 ± 9.897	56.51 ± 8.124	1.692	0.189
Gender (male/female)^b^	30/8	32/12	31/10	0.428	0.807
Education (years)^a^	11.72 ± 3.504	11.67 ± 4.209	12.00 ± 3.388	0.060	0.942
NIHSS^c^	4 (4)	3 (3)	–	391.500	0.107
BI^c^	65 (38)	90 (35)	–	422.500	0.230
FMA^c^	79 (30)	85 (16)	–	419.500	0.223
MoCA^d^	19.38 ± 4.875	20.06 ± 6.228	–	−0.455	0.651
SDS^c^	48 (4)	47 (7)	–	130.500	0.097

^a^*P*-values were obtained by one-way ANOVA.

^b^*P*-value for gender distribution in the three groups was obtained by the chi-square test.

^c^The *P*-values were obtained using Mann–Whitney U test.

^d^The *P*-values were obtained using independent sample *t*-test. LBGIS, left basal ganglia ischemic stroke; RBGIS, right basal ganglia ischemic stroke; HC, healthy control; NIHSS, National Institute of Health stroke scale; BI, Barthel Index; FMA, Fugl-Meyer assessment; MoCA, Montreal cognitive assessment; SDS, self-rating depression scale.

### Comparison of VMHC values between LBGIS group, RBGIS group and HC group

**FIGURE 2:**
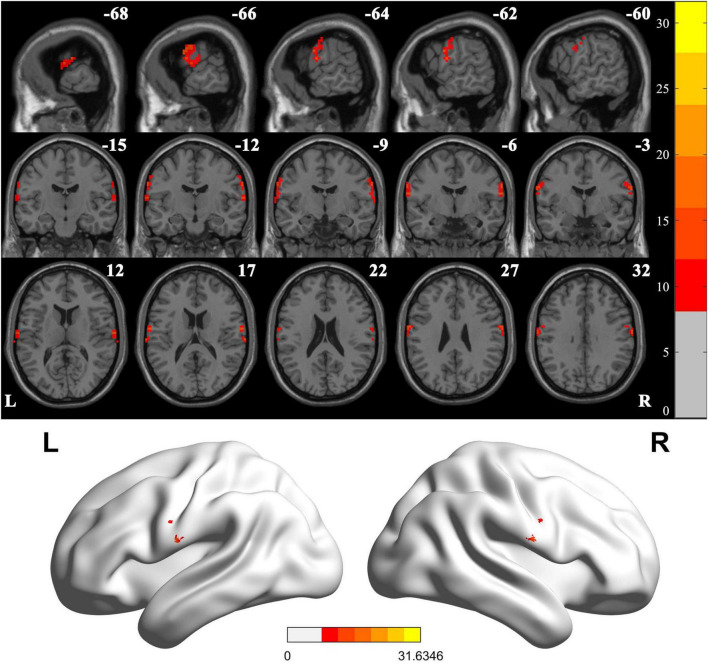
Voxel-mirrored homotopic connectivity differences across groups. One-way analysis of variance shows brain regions with significant differences of the VMHC values across the three groups (corrected with GRF for multiple comparisons: voxel *P* < 0.001, cluster *P* < 0.05). GRF, Gaussian random field. L, Left; R, Right.

**FIGURE 3:**
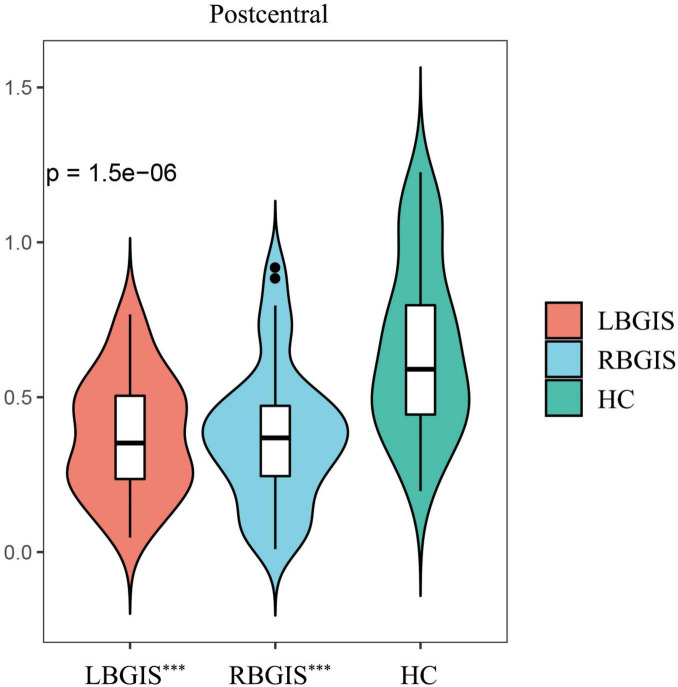
*Post hoc* pairwise comparisons showed that compared with HC group, LBGIS and RBGIS groups showed decreased VMHC value in postcentral gyrus. LBGIS, left basal ganglia ischemic stroke; RBGIS, right basal ganglia ischemic stroke; HC, healthy control. ***p < 0.001.

When comparing VMHC values in these three groups using one-way ANOVA, significant differences were detected in postcentral gyrus, extending to precentral gyrus (Figure 2 and Table 2; Voxel *P* < 0.001, Cluster *P* < 0.05, GRF correction) (Eklund et al., 2016). *Post hoc* analysis showed that in the pairwise ROI-based comparison, individuals with LBGIS and RBGIS exhibited reduced VMHC values compared to HC groups. There was no significant difference between the LBGIS and RBGIS groups (Figure 3 and Table 3).

**TABLE 2:** Brain regions with significant differences in VMHC.

Brain region (AAL)	Brain regions (Brodmann)	Peak MNI coordinates			Number of voxels	Peak *F* values
		X	Y	Z		
Postcentral^a^	BA43	± 66	−9	30	79	14.6767

^a^The peak value of the clusters was in the postcentral gyrus, the clusters also covered part of the precentral gyrus. Postcentral, postcentral gyrus.

**TABLE 3:** *Post hoc* pairwise comparisons of the mean VMHC z-score in three groups.

Brain region	LBGIS	RBGIS	HC	*Post hoc P*-value		
				LBGIS vs. HC	RBGIS vs. HC	LBGIS vs. RBGIS
Postcentral ^a^	0.37 ± 0.19	0.38 ± 0.22	0.65 ± 0.28	< 0.001***	< 0.001***	0.98

^a^The peak value of the clusters was in the postcentral gyrus, the clusters also covered part of the precentral gyrus. LBGIS, left basal ganglia ischemic stroke; RBGIS, right basal ganglia ischemic stroke; HC, healthy control; Postcentral, postcentral gyrus.

***p < 0.001.

### Correlation analysis between VMHC values of brain regions with significant differences and clinical scores

**FIGURE 4:**
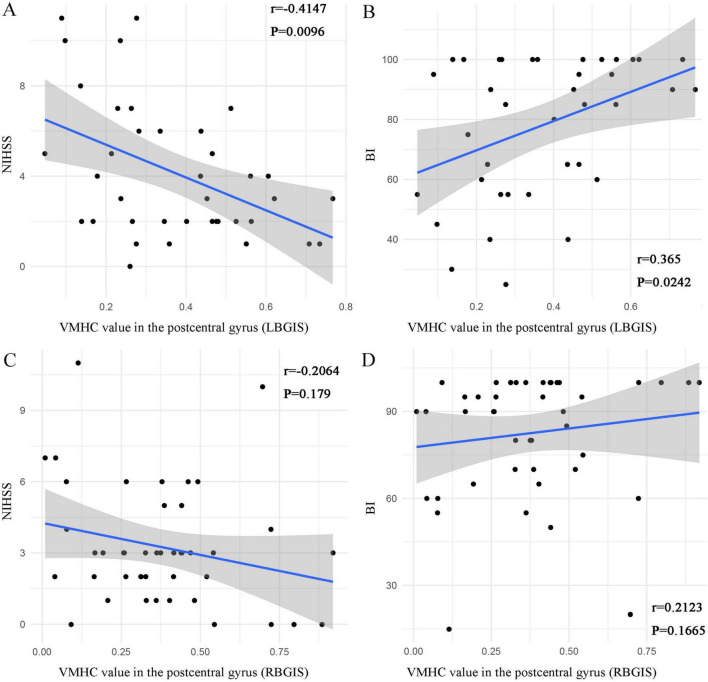
Scatter plots depict the relationship between VMHC values in abnormal regions and clinical variables in BGIS patients. In the LBGIS group, the VMHC values of the postcentral gyrus was negatively correlated with NIHSS [**(A)** *r* = –0.4147, *P* = 0.0096] and positively correlated with BI [**(B)** *r* = 0.365, *P* = 0.0242] **(A,B)**. In the RBGIS group, no significant correlations were detected between VMHC values and NIHSS (*r* = –0.2064, *P* = 0.179) and BI (*r* = 0.2123, *P* = 0.1665) **(C,D)**.

In the LBGIS group, the VMHC values showed a negative correlation with NIHSS (Spearman correlation analysis, *r* = −0.4147, *P* = 0.0096) and a positive correlation with BI (Spearman correlation analysis, *r* = 0.365, *P* = 0.0242) (Figures 4A, B). However, no significant correlations were detected between VMHC values and NIHSS (Spearman correlation analysis, *r* = −0.2064, *P* = 0.179) and BI (Spearman correlation analysis, *r* = 0.2123, *P* = 0.1665) in the RBGIS group (Figures 4C, D).

## Discussion

VMHC is a method used to assess the synchronization of spontaneous activity in homotopy regions of the brain between hemispheres. It is predominantly employed in psychiatric and neuroscience research to investigate brain function by examining activity patterns in symmetrical regions of the left and right hemispheres of the brain. For instance, in rs-fMRI studies involving patients with major depression, schizophrenia, Alzheimer’s disease spectrum disorder, and early Parkinson’s disease, VMHC has been utilized to compare interregional communication and coordination, identify analogous diseases, and characterize the severity of certain conditions (Chen et al., 2024; Hu et al., 2024; Liu et al., 2024).

In this study, VMHC method was used to investigate the abnormal connectivity of homologous regions in the whole brain of patients with acute BGIS. Our study demonstrated that individuals with LBGIS and RBGIS exhibited impaired interhemispheric synchronization in the postcentral gyrus and precentral gyrus when compared to HC individuals. The postcentral gyrus and precentral gyrus are integral to the processing and regulation of sensation and movement. The basal ganglia serve as a critical hub within the pyramidal tract and sensory conduction pathways, and dysfunction within this pathway is a well-established mechanism underlying motor and sensory deficits in ischemic stroke. Consequently, the observed bilateral incoordination of the anterior and posterior central gyrus may be associated with the occurrence of these deficits. These regions are integral components of the sensorimotor network (SMN), responsible for motor control and somatosensory processing (Carter et al., 2010). It is postulated that following BGIS, there is a reorganization of local brain function and a reduction in interhemispheric communication, leading to diminished connectivity between the bilateral SMNs.

The specific mechanism of the decrease of VMHC value after BGIS may be related to the remodeling of brain function. Prior research has indicated a reduction in functional connectivity within homotopic brain regions among individuals with chronic stroke in comparison to those without neurological impairment. Specifically, individuals with chronic stroke exhibit diminished values of VMHC in various brain regions, including the central anterior gyrus and central posterior gyrus in the SMN when compared to healthy controls (Tang et al., 2016). A separate investigation involving individuals with subacute stroke found that, in comparison to a control group of healthy individuals, those with subacute stroke exhibited significantly decreased VMHC values in the superior frontal gyrus and central anterior gyrus. A negative correlation was observed between the FMA score in the central anterior gyrus and the dynamic variability of VMHC (Chen et al., 2021). Furthermore, findings from a longitudinal study indicate that individuals with subcortical ischemic motor disorders exhibit notably reduced VMHC values in regions associated with movement-related functions, such as the prefrontal lobe, parahippocampal area, central anterior gyrus, supplementary motor area, and middle frontal gyrus, when compared to individuals without such disorders. Specifically, the VMHC values in the upper prefrontal lobe may serve as a reliable indicator for distinguishing stroke patients from healthy individuals (Li et al., 2022). Considering the strong correlation between NIHSS score and FMA score, especially in BGIS patients, we considered that our study yielded comparable findings to prior researches, specifically demonstrating decreased VMHC values in the precentral and postcentral gyrus among patients with BGIS.

Correlation analysis shows that, in LBGIS group, the mean VMHC value in these regions displayed a negative correlation with NIHSS scores and a positive correlation with BI scores. These findings suggest that the aberrant neural function in these areas of LBGIS patients is associated with physical motor dysfunction. In subsequent research, transcranial magnetic stimulation targeting these areas may prove to be a significant focus for rehabilitation. Nevertheless, the absence of a correlation between mean VMHC values in the RBGIS patient cohort and clinical scale scores raises questions regarding the anticipated outcomes. This discrepancy may be attributed to distinct neural remodeling patterns in RBGIS patients compared to LBGIS patients, or variations in sample characteristics, including disease severity and lesion size. Further investigation is warranted to elucidate the neural mechanisms underlying VMHC desynchronization in bilateral BGIS patients.

The findings also suggest that despite variations in lesion location and resulting motor impairments, there is no discernible functional disparity between the two groups. Furthermore, the study confirms the absence of significant functional distinctions in the impact of left and right BGIS on remote brain areas. The statistically significant differences in VMHC values between patients with left and right BGIS compared to those in the HC group suggest that bilateral interhemispheric connections are compromised following the onset of basal ganglia infarction, irrespective of lesion location.

In summary, from a clinical point of view, this study contributes to a deeper understanding of the changes in cerebral interhemispheric functional connectivity after ischemic stroke in the basal ganglia and its relationship with neurological impairment. In practical sense, this research can provide scientific basis for making individual rehabilitation plan. For example, by analyzing changes in functional connections between hemispheres, it is possible to better understand which brain connections are more critical for specific rehabilitation exercises. In addition, such research may also reveal new targets for rehabilitation interventions, such as promoting the recovery of neural function by modulating specific brain region connections.

Our research is subject to various constraints. Firstly, the stringent criteria for participant selection and the division of hemispheres resulted in relatively small sample sizes for each group, necessitating larger sample sizes in future studies to validate the findings. Secondly, the study was cross-sectional in nature and did not monitor the dynamic changes in VMHC in patients with BGIS. Longitudinal data in future research will provide a deeper insight into the neuroimaging mechanisms of BGIS.

## Conclusion

This study found, based on the analysis of VMHC technique using rs-fMRI, that patients with unilateral BGIS showed brain functional remodeling characterized by reduced functional synchronization and coordination between SMNs of both hemispheres, which may be involved in the neurological mechanism of motor function impairment in these patients.

## Data Availability

The original contributions presented in this study are included in this article/supplementary material, further inquiries can be directed to the corresponding authors.
